# Isolation and Characterization of Two Novel Bacteria *Afipia cberi* and *Mesorhizobium hominis* from Blood of a Patient Afflicted with Fatal Pulmonary Illness

**DOI:** 10.1371/journal.pone.0082673

**Published:** 2013-12-18

**Authors:** Shyh-Ching Lo, Bingjie Li, Guo-Chiuan Hung, Haiyan Lei, Tianwei Li, Jing Zhang, Kenjiro Nagamine, Shien Tsai, Mark J. Zucker, Ludmilla Olesnicky

**Affiliations:** 1 Tissue Microbiology Laboratory, Division of Cellular and Gene Therapies, Office of Cellular, Tissue and Gene Therapies, Center for Biologics Evaluation and Research, Food and Drug Administration, Bethesda, Maryland, United States of America; 2 Department of Medicine, Newark Beth Israel Medical Center, Newark, New Jersey, United States of America; 3 Department of Pathology, Newark Beth Israel Medical Center, Newark, New Jersey, United States of America; University of Iowa Carver College of Medicine, United States of America

## Abstract

We recently isolated and discovered new *Bradyrhizobiaceae* microbes from the cryopreserved culture broth of blood samples from 3 patients with poorly defined illnesses using modified SP4 media and culture conditions coupled with genomic sequencing. Using a similar protocol, we studied a previously cryopreserved culture broth of blood sample from a patient who had succumbed to an acute onset of fulminant pulmonary illness. We report that two phases of microbial growth were observed in the re-initiated culture. Biochemical and genomic characterization revealed microbes isolated from the first phase of growth were new *Afipia* species of *Bradyrhizobiaceae*, tentatively named *A. cberi* with a ~ 5 MB chromosome that was different from those of all previously known *Afipia* microbes including the newly discovered *A. septicemium*. The microbes isolated from the second phase of growth were prominent sugar assimilators, novel *Phyllobacteriaceae*, phylogenetically most closely related to *Mesorhizobium* and tentatively named *M. hominis* with a ~ 5.5 MB chromosome. All *A. cberi* isolates carry a circular ~ 140 KB plasmid. Some *M. hominis* isolates possess a circular ~ 412 KB plasmid that can be lost in prolonged culture or passage. No antibiotics resistant genes could be identified in both of the *A. cberi* and *M. hominis* plasmids. Antibiotic susceptibility studies using broth culture systems revealed isolates of *A. cberi* could be sensitive to some antibiotics, but all isolates of *M. hominis* were resistant to essentially all tested antibiotics. However, the cell-free antibiotics susceptibility test results may not be applicable to clinical treatment against the microbes that are known to be capable of intracellular growth. It remains to be determined if the 2 previously unknown *Rhizobiales* were indeed pathogenic and played a role in the pulmonary disease process in this patient. Specific probes and methods will be developed to re-examine the diseased lungs from patient's autopsy.

## Introduction

The isolation of microbial pathogens by culture is crucial for clinical diagnosis in medical microbiology; however, microbial culture systems developed in the laboratory do not truly replicate the complex and dynamic properties of various tissues in human body. Some microbes fail to proliferate or grow to a detectable concentration in the axenic cultures used in the laboratory to grow and isolate the microbes. The newly developed next generation sequencing (NGS) technology has provided a revolutionary tool for the field of metagenomics through identification of unknown infectious microbes in a “culture-free” or “uncultured” condition [1,2]. The rapid advancements of NGS were used successfully in detecting and analyzing very low numbers of gene sequences of previously uncultivated microbes in an examined sample [3–5]. 

The new microbial detection capabilities of NGS technology have prompted us to re-examine some of our cryopreserved broths of blood cultures that were suspected of having low numbers of unknown, inactive microbes despite negative result of microbial isolation. These blood samples were previously submitted to the Laboratory of Infectious Diseases Pathology at Armed Forces Institute of Pathology (AFIP) for diagnostic consultations [6] and detection of possible unusual infections, such as mycoplasmal agents [7,8]. Encouraged by our recent success of isolating novel *Afipia septicemium* and identifying previously unknown *Bradyrhizobium* sp. OHSU-III from the cryopreserved SP4 broth of blood cultures from 3 patients with poorly defined illnesses [9], we decided to examine the cryopreserved SP4 culture broth from one particular patient’s blood sample submitted from Newark Beth Israel Medical Center (NBIMC) in 1999.  The blood sample was obtained from a previously healthy 36 year-old white male right before he succumbed to an acute onset of a fulminant pulmonary illness. This highly unusual case with subsequent autopsy examination was studied extensively at NBIMC and AFIP without successfully identifying any etiological agent. The cause of death was reported to be necrotizing pneumonitis of presumably viral origin. 

In this study we report isolation of 2 previously unknown *Rhizobiales* bacteria in the re-initiated blood-sample culture from this patient. Metabolic and genomic characterizations revealed that microbes isolated from the 1^st^ phase of growth (NBIMC_P1) in culture were new *Afipia* *sp.* in the Family of *Bradyrhizobiaceae* and microbes isolated from the 2^nd^ phase of growth (NBIMC_P2) in culture were new *Phyllobacteriaceae*, most closely related to those of *Mesorhizobium* *sp*. We have also examined antibiotics susceptibilities of the 2 novel microbes isolated from the patient blood.

## Materials and Methods

Studying previously frozen cultures of the blood samples was conducted under FDA Research Involving Human Subjects Committee (RIHSC) protocol 10-008B entitled "Detection of Infectious Agents in Previously Frozen Blood Samples from Patients with Various Illnesses and Healthy Blood Donors". The original clinical presentations of patients were provided by Dr. Zucker in this study after the IRB of NBIMC and Bamabas Health Corporate legal representatives reviewed and determined that the activity did not meet the definition of human subject research per 45 CFR 46.102(d), a written informed consent from the patient was waived. The clinical illness was described in the study without revealing the patient’s identity. 

### Blood sample, patient and clinical presentation

A 36-year old previously healthy white male woke up with a headache and fever after playing golf the day before. The patient went to see his primary care physician who prescribed the antibiotic “levofloxacin”. The patient did not improve clinically and developed a macular rash. His physician changed the antibiotics to “amoxicillin/clavulanic acid”. Despite the antibiotics, the patient’s pulmonary status worsened with the development of bilateral pulmonary infiltrates. He was admitted to the local medical center hospital, was intubated, and within 5 days developed acute respiratory distress syndrome (ARDS). He was then transferred to Newark Beth Israel Medical Center (NBIMC) for further evaluation and treatment. Extracorporeal membrane oxygenation (ECMO) was initiated. Broad spectrum antibiotics targeting both bacteria and Rickettsia were begun and additional laboratory studies were sent to the local laboratory and to the AFIP in the hope of identifying an infectious agent. Despite the ECMO and the antibiotics, the patient’s clinical condition progressively deteriorated and he expired 2 weeks after admission to NBIMC. An autopsy was conducted in less than 4 hours. However, no etiologic cause was identified. The AFIP pathology report also concluded that no infectious agent was identified.

### Broth medium and agar plates

Preparation of the modified SP4 medium with new supplements and other microbial broths as well as agar plates was previously described in detail [9].

### Microbial cultures

#### (1): Original microbial cultures for diagnostic study

The laboratory received 600 to 700 uL partially hemolyzed whole blood (WB) for the diagnostic study in 1999. Details of protocol for of isolation of mycoplasmal agents were previously described [7]. For this particular sample, 100 µL of WB were inoculated into a culture tube containing 5 mL SP4 broth medium supplemented with 10% fetal bovine serum (FBS). In addition, 50 µL of WB were streaked on an individual SP4 agar plate and BHI agar plate. Four separate sets of SP4 broth cultures and agar plates were set up and kept respectively in either an aerobic condition or in GasPack, at 37 °C or at 32 °C. For each set of cultures set up, 2 (negative) control cultures containing the same amount of broth without inoculation of the specimen to be examined were included and studied in parallel. Aliquots of broth were routinely streaked on SP4 agar plates and BHI agar plates and were tested by PCR assays using mycoplasma-specific primers at different intervals of sample cultivation during the months-long follow-up. The broth samples were also routinely examined for color changes and by dark-field/phase-contrast microscopy for evidence of microbial growth. At the end of diagnostic study, the SP4 broth of the culture kept at 32 °C in GasPack was suspected of having a low number of unknown microbes and was mixed with glycerol (final ~ 10%) and cryopreserved at -80 °C. 

#### (2): Re-initiated microbial cultures for microbial isolation

The procedures of re-initiating previously cryopreserved SP4 broth culture and examination for evidence of microbial growth were described in detailed [9]. Briefly, cryopreserved SP4 broth (100 to 200 μL) containing cultures established from blood samples were inoculated into 25 cm^2^ tissue culture flasks containing ~ 7 mL of modified SP4 medium supplemented with 10% irradiated FBS. Three sets of cultures were kept separately at room temperature (RT, ~ 25 °C), 30 °C and 35 °C. For each set of cultures, at least 2 control cultures containing the same modified SP4 broth medium and FBS were inoculated with frozen plasma and 0.3 X 10^6^ of PBMC from healthy blood donors and studied in parallel. None of the control cultures showed microbial growth in the study.

### Biochemical and metabolic study using Bio-Log ID system, electron microscopy study, whole genome sequencing, 16S rRNA gene and rDNA operon comparison and phylogenetic analysis

The studies were conducted as previously described [9]. The GenBank Accession Numbers for NBIMC_P1-C1, NBIMC_P1-C2, NBIMC_P1-C3, NBIMC_P2-C1, NBIMC_P2-C2, NBIMC_P2-C3 and NBIMC_P2-C4 are AVBK00000000, AVBL00000000, AVBM00000000, AVBN00000000, AVBO00000000, AVBP00000000 and AVBQ00000000, respectively.

### Construction of draft genomes and genome content comparison

Alignment of the formed contigs assembled from genomic sequencing into draft genomes for *Afipia* sp. NBIMC_P1 isolates was previously described in detail [9]. The complete genome of *Mesorhizobium loti* (GenBank accession number NC_002678.2) was used as a reference genome in draft genome construction for NBIMC_P2 isolates. As described in the previous study [9], the schematic sequence alignment for all input draft genome sequences using CONTIGuator [10] and comparison of dissimilarity for genome contents among the related microbes were performed using progressiveMauve [11]. 

### Antibiotic susceptibility test

Antibiotic susceptibility tests for each antibiotic were performed in triplicates using marcobroth dilution method [12] in 24-well plates. Logarithmically growing bacteria (1-5 × 10^5^ c.f.u/mL, final concentration) were inoculated into each well of 2 mL of YM broth containing ranged concentrations of each antibiotic tested. MIC (minimum inhibition concentration) was read on the plates incubated at 30 °C for 72 h and 48 h for *Afipia* *sp.* isolates and *Mesorhizobium* *sp.* isolates, respectively. *Escherichia coli* (*E. coli*) ATCC 25922 and *Staphylococcus aureus* (*S. aureus*) ATCC 29213 were studied in parallel as the quality control for the antibiotics potency. All antibiotics tested in this study were purchased from Sigma-Aldrich Co. (St. Louis, MO). 

## Results

### Growth and isolation of microbes from cryopreserved cultures of NBIMC blood sample

**Figure 1:**
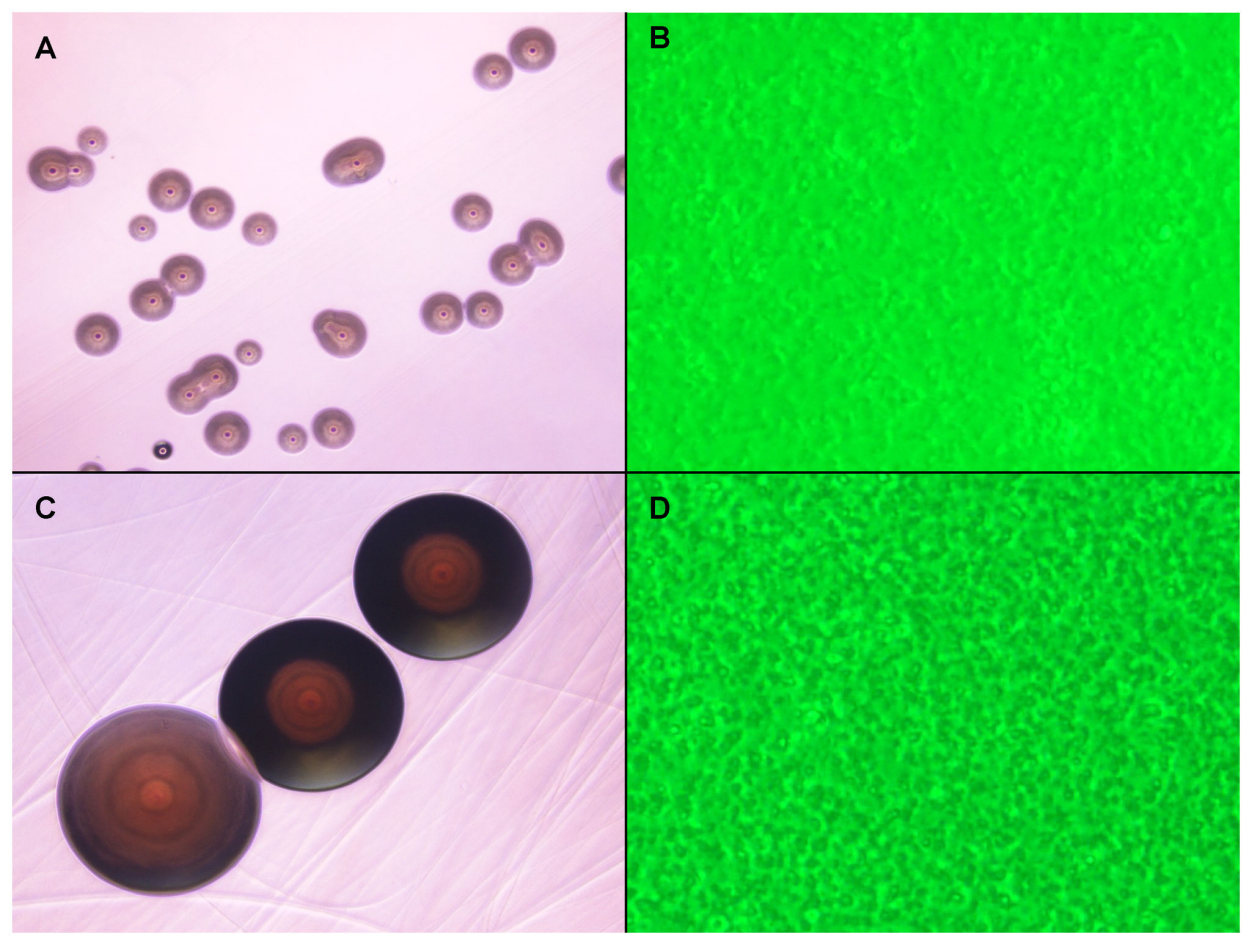
Photomicrographs of NBIMC_P1 and NBIMC_P2 microbes. **A**: Photomicrograph of NBIMC_P1-C2 microbes forming microscopic colonies on the surface of YM agar plate after 2 weeks of incubation at room temperature (RT, ~ 25 °C). The streaked lines on the agar plate could be identified. 40X. **B**: Photomicrographs of NBIMC_P1-C2 microbes growing in modified SP4 broth culture kept at RT after 10 days. The microbes stayed at the bottom of undisturbed culture flasks. Many of the NBIMC_P1 C2 microbes aggregated into clumps and adhered on the plastic flask surface. Phase contrast with green filter. 400X . **C**: Photomicrograph of NBIMC_P2-C1 forming microscopic colonies on the surface of YM agar plate after 1 week of incubation at room temperature (RT, ~ 25 °C). The streaked lines on the agar plate could be easily seen. 40X. **D**: Photomicrograph of NBIMC_P2-C1 microbes growing in modified SP4 broth culture kept at RT after 10 days. The microbes grew into much higher density and produced turbidity in the culture. Many microbes formed aggregates and adhered on the plastic flask surface. Phase contrast 400X.

The re-initiated blood cultures using modified SP4 broth kept separately at room temperature (RT, ~ 25 °C), 30 °C and 35 °C were examined under an inverted microscope 2 to 3 times a week. The cultures initially showed no sign of microbial growth. The broths of the cultures streaked on SP4-agar and BHI-agar plates also produced no detectable colonies. However, cultures kept at RT began to show sign of microbial growth after ~ 10 weeks. The cultures were diluted 1:3 with fresh modified SP4 media and expanded into 3 flasks. The microbes in these cultures gradually grew into a plateau containing higher cell density with low, but detectable turbidity in the broths after additional 3 to 4 weeks. Gram staining revealed the microbes in the broths were Gram-negative bacteria. The broths of the cultures streaked on SP4-agar plates also started to produce microscopic colonies. The colonies could be detected under microscope on the agar plates kept at RT for ~ 1-2 weeks (Figure 1 A). Individual microscopic colonies were picked with care and grown in cultures using modified SP4 broth media with or without supplement of FBS at RT (Figure 1B). The colony-derived microbes adapted well enough to grow in YM broth without supplement of FBS. The bacteria derived from individual colonies picked on agar plate (NBIMC_P1-C1, P1-C2 and P1-C3) were studied for biochemical reactions and metabolic property testing as well as whole-genome sequencing. None of the control cultures set up with the same SP4 broth containing irradiated FBS and followed in parallel showed evidence of microbial growth in the study.

Unexpectedly, in the continual follow-up, the SP4 broth cultures kept at RT gave signs of having an apparent 2^nd^ phase of microbial growth ~ 5 weeks after microbes were isolated from the initial phase (Phase 1, P1) of growth. Growth of microbes in the 2^nd^ phase (Phase 2, P2) reached a significantly higher plateau of cell density than that in the P1, produced more prominent turbidity and acidity color change in the broth. Gram staining revealed the more rapidly proliferating microbes in the cultures were also Gram-negative bacteria. The culture broths streaked on SP4 agar produced colonies clearly larger than those formed previously from microbes of the 1^st^ phase of growth (Figure 1C). The colonies were detectable after 5-7 days by the naked eye appearing to be lightly yellowish or brownish in color. Individual colonies were again picked and grown at RT in broth cultures using modified SP4 broth media, with or without supplement of FBS (Figure 1D). The bacteria derived from the selected colonies of Phase 2 growth (NBIMC_P2-C1, P2-C2, P2-C3 and P2-C4) were also examined for biochemical properties and by whole-genome sequencing. As described in the Materials/Methods, for each sample-testing culture, at least 2 negative control cultures were set up in parallel using the same culture media and kept together at each respective temperature and culture conditions. None of these control cultures grew any microbes.

### Biochemical characterization using Bio-Log identification system

NBIMC_P1-C1, P1-C2 and P1-C3 cloned from the Phase 1 growth could grow on BCYE agar, YM agar or SP4 agar kept at both 30 °C and 35 °C. The microbes could also grow on agar plates made using tissue culture medium RPMI-1640 with 5% fetal bovine serum at 37 °C in a CO_2_ incubator. In comparison, NBIMC_P2-C1, P2-C2, P2-C3 and P2-C4 cloned from the Phase 2 growth could only grow on YM and BCYE agars kept at 32 °C (BCYE agars at 33 °C) and did not grow well on these agars kept at 35 °C (Table 1). The microbes obtained from both the 1^st^ and the 2^nd^ phases of growth in the culture could not grow on MacConkey agar kept at any temperature tested. Biochemical properties and metabolic characterization of representative single-colony cloned were studied using Biolog bacterial identification system. The system offers phenotype microarrays that enable microbes to be evaluated for thousands of phenotypes under thousands of culture conditions. In the study, all bacteria isolates were first grown on YM or BCYE agar plates for 7 days at 30 °C and then transferred to the Biolog micro-plates for metabolic testing. Most significantly, results of the studies for sugar assimilations by the microbes including glucose, arabinose, mannose, mannitol and N-acetyl glucosamine showed that all the isolates/clones from the 1^st^ phase growth tested negative and all the isolates/clones from the 2^nd^ phase growth tested positive (Table 1; [13,14]). However, the metabolic test results of the isolates obtained from both the 1^st^ and the 2^nd^ phases (NBIMC_P1 and NBIMC_P2) of growth in the culture did not match well with any specific bacterium in the Biolog identification database. The biochemical characterization study produced no identification match for NBIMC_P1 isolates in the database. The study for NBIMC_P2 isolates had a partial match of 33% to 50% similarity with *Aminobacter aminovorans*, in the Family of *Phyllobacteriaceae*, Order of *Rhizobiales*.

**Table 1:** Comparison of growth characteristics, biochemical reactions and G+C contents of NBIMC_P1 isolates and NBIMC_P2 isolates.

**Tests**	***Afipia cberi***			***Mesorhizobium hominis***				**Studies Results of References**					
	**NBIMC_P1**			**NBIMC_P2**				***Amino bacteraminovorans#***	Mesorhizobium loti*	Mesorhizobium amorphae*	Bradyrhizobium japonicum*	***Agrobacterium radiobacter***	
	**C1**	**C2**	**C3**	**C1**	**C2**	**C3**	**C4**						
**Motility:**													
**Growth on:**													
**BCYE agar** (**RT**)	**P**	**P**	**P**	**P**	**P**	**P**	**P**		**P**	**P**	**P**	**P**	
**30 °C**	**P**	**P**	**P**	**P**	**P**	**P**	**P**		**P**	**P**	**P**	**P**	
**32 °C**	**P**	**P**	**P**	**P**	**P**	**P**	**P**		**P**	**P**	**P**	**P**	
**33 °C**	**P**	**P**	**P**	**P**	**P**	**P**	**P**		**P**	**P**	**P**	**P**	
**35 °C**	**P**	**P**	**P**	**N**	**N**	**N**	**N**		**P**	**N**	**N**	**P**	
**37 °C**	**N**	**N**	**N**	**N**	**N**	**N**	**N**		**N**	**N**	**N**	**N**	
**YM agar** (**RT**)	**P**	**P**	**P**	**P**	**P**	**P**	**P**		**P**	**P**	**P**	**P**	
**30 °C**	**P**	**P**	**P**	**P**	**P**	**P**	**P**		**P**	**P**	**P**	**P**	
**32 °C**	**P**	**P**	**P**	**P**	**P**	**P**	**p**		**P**	**P**	**P**	**P**	
**33 °C**	**P**	**P**	**P**	**N**	**N**	**N**	**N**		**P**	**P**	**N**	**P**	
**35 °C**	**P**	**P**	**P**	**N**	**N**	**N**	**N**		**P**	**N**	**N**	**P**	
**37 °C**	**N**	**N**	**N**	**N**	**N**	**N**	**N**		**N**	**N**	**N**	**N**	
**Columbia agar_5% SB (30 °C**)	**N**	**N**	**N**	**P**	**P**	**P**	**P**						
**32 °C**	**N**	**N**	**N**	**P**	**P**	**P**	**P**						
**33 °C**	**N**	**N**	**N**	**N**	**N**	**N**	**N**						
**34 °C**	**N**	**N**	**N**	**N**	**N**	**N**	**N**						
**Chocolate agar** (**RT**)	**N**	**N**	**N**	**P**	**P**	**P**	**P**						
**30 °C**	**N**	**N**	**N**	**P**	**P**	**P**	**P**						
**35 °C**	**N**	**N**	**N**	**N**	**N**	**N**	**N**						
**BHI agar** (**RT**)	**N**	**N**	**N**	**P**	**P**	**P**	**P**						
**30 °C**	**N**	**N**	**N**	**P**	**P**	**P**	**P**						
**32 °C**	**N**	**N**	**N**	**p**	**p**	**p**	**p**						
**33 °C**	**N**	**N**	**N**	**N**	**N**	**N**	**N**						
**RPMI-5% FBS 35 °C/CO2 incubator**	**P**	**P**	**P**	**N**	**N**	**N**	**N**						
**37 °C/CO2 incubator**	**P**	**P**	**P**	**N**	**N**	**N**	**N**						
**MacConkey agar** (**RT**)	**N**	**N**	**N**	**N**	**N**	**N**	**N**						
**30°C**	**N**	**N**	**N**	**N**	**N**	**N**	**N**						
**35 °C**	**N**	**N**	**N**	**N**	**N**	**N**	**N**						
**Nitrate reduction**	**p**	**N**	**N**	**N**	**N**	**N**	**N**	**N**	**P**	**P**	**P**	**P**	
**Catalase**	**p**	**p**	**p**	**p**	**p**	**p**	**p**	**P**	**P**	**p**	**P**	**P**	
**Oxidase**	**P**	**P**	**P**	**P**	**P**	**P**	**P**	**P**	**P**	**p**	**P**	**p**	
**Assimilation of**:													
**Glucose**	**N**	**N**	**N**	**P**	**P**	**P**	**P**	**P**	**N**			**P**	
**Arabinose**	**N**	**N**	**N**	**P**	**P**	**P**	**P**	**P**	**P**	**P**	**N**	**P**	
**Mannose**	**N**	**N**	**N**	**P**	**P**	**P**	**P**		**N**			**P**	
**Mannitol**	**N**	**N**	**N**	**P**	**P**	**P**	**P**	**P**	**N**			**P**	
**N-Acetylglucosamine**	**N**	**N**	**N**	**P**	**P**	**P**	**P**		**N**			**P**	
**Gluconate**	**p**	**p**	**p**	**P**	**P**	**P**	**P**	**N**	**p**			**P**	
**Malate**	**N**	**p**	**p**	**N**	**N**	**N**	**N**		**N**			**p**	
**D-Malic acid**	**N**	**N**	**N**	**N**	**N**	**N**	**N**		**N**			**N**	
**L-Malic acid**	**N**	**p**	**p**	**N**	**N**	**N**	**N**	**N**	**N**			**p**	
**Citrate**	**N**	**N**	**N**	**N**	**N**	**N**	**N**		**N**			**N**	
**Phenylacetate**	**P**	**P**	**P**	**p**	**N**	**p**	**p**		**N**			**N**	
**Raffinose**	**N**	**N**	**N**	**N**	**N**	**N**	**N**	**P**	**P**	**P**	**N**	**N**	
**Lactose**	**N**	**N**	**N**	**N**	**N**	**N**	**N**		**P**	**P**	**N**	**P**	
**Melibiose**	**N**	**N**	**N**	**N**	**N**	**N**	**N**		**P**	**P**	**P**	**P**	
**D-Galactose**	**N**	**N**	**N**	**N**	**N**	**N**	**N**		**P**	**P**	**N**	**P**	
**L-Rhamnose**	**N**	**N**	**N**	**P**	**P**	**P**	**P**	**P**	**P**	**P**	**N**	**P**	
**Maltose**	**N**	**N**	**N**	**P**	**P**	**P**	**P**	**P**	**P**	**P**	**N**	**P**	
**D-Trehalose**	**N**	**N**	**N**	**P**	**P**	**P**	**P**	**N**	**P**	**P**	**N**	**P**	
**Sucrose**	**N**	**N**	**N**	**P**	**P**	**P**	**P**	**P**	**P**	**P**	**N**	**P**	
**D-Cellobiose**	**N**	**N**	**N**	**P**	**P**	**P**	**P**	**P**					
**D-Sobitol**	**N**	**N**	**N**	**P**	**P**	**P**	**P**	**N**					
**Propionic acid**	**N**	**N**	**N**	**N**	**N**	**N**	**N**	**P**					
**Biolog ID**:	**N/A**	**N/A**	**N/A**	***Aminobacter aminovorans***									
**(% similarity)**				**33%**	**43%**	**38%**	**50%**						
**G+C content (%)**	**61.5%**	**61.5%**	**61.5%**	**63.4%**	**63.4%**	**63.4%**	**63.4%**						

P = positive, p = weak positive, N = negative, N/A = not available

^#^ International Journal of Systematic and Evolutionary Microbiology (2005), 55, 1827–1832 (Ref 13)

^*^ International Journal of Systematic and Evolutionary Microbiology (2001), 51, 1011–1021 (Ref 14)

### Ultrastructure study of microbial isolates from the 1^st^ and the 2^nd^ phases of growth in culture

**Figure 2:**
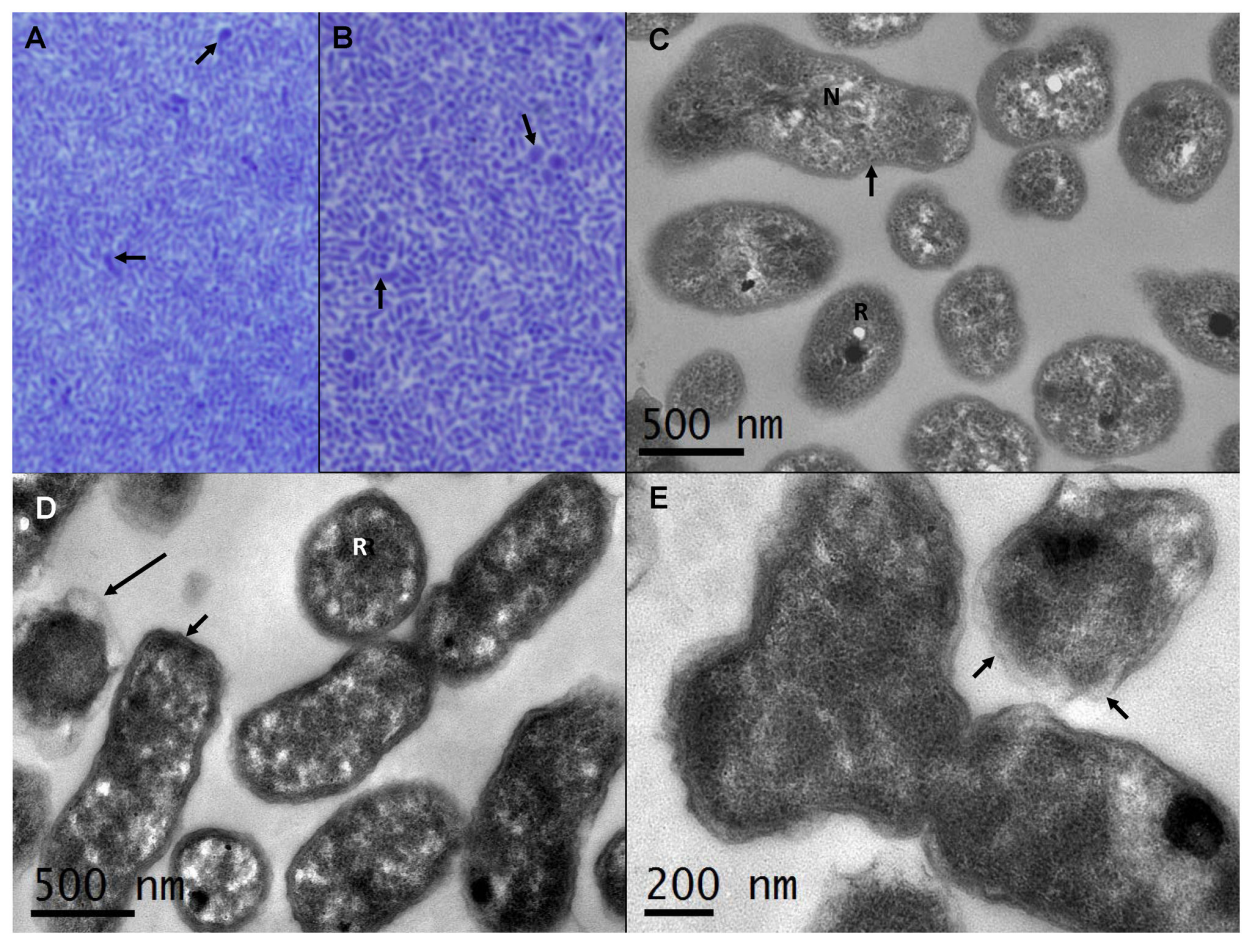
Thick section photomicrographs and ultrathin electron micrographs of NBIMC_P1 and NBIMC_P2 microbes. **A** and **B**: Thick section photomicrographs of NBIMC_P1-C1 and NBIMC_P2-C2 cultures using modified SP4 broth. The section of concentrated NBIMC_P1-C1 reveals slender pointed microbes (A). The section of concentrated NBIMC_P2-C2 reveals slightly larger or thicker rods (B). Both sections show some microbes (arrows) that are more polymorphic in shape and larger in size with longitudinal and cross sectioning. The concentrated microbes were fixed with 2.5 % glutaraldehyde, post-fixed with 1% osmium tetroxide and embedded in epoxy resin. The thick sections were stained using 1% toluene blue. 1000X. **C** and **D**: Electron photomicrographs of NBIMC_P1-C1 (C) and NBIMC_P2-C2 (D) captured in ultrathin sections. Typical Gram-negative bacteria wall structure (short arrows), intracellular ribosomal structures (R), electron-dense bodies and nucleic acid (N) as well as scale bars are indicated for both sections. Atypical wall structures with loose and wavy outer membranes are seen in some of NBIMC_P2-C2 microbes (long arrow). The ultrathin sections were stained with uranyl acetate and lead citrate. **E**: Electron micrograph of NBIMC_P2-C2 microbes that are more polymorphic in shape captured at higher magnification. External membrane (arrow) of the Gram-negative bacteria wall structure appeared to be less rigid and wavy. The scale bar is indicated.

The single-colony cloned bacteria from culture broth showing the 1^st^ phase of microbial growth (NBIMC_P1-C2) and single-colony cloned bacteria from culture broth showing the 2^nd^ phase of microbial growth (NBIMC_P2-C1) were subjected for ultra-structure study by electron microscopy. Both of the bacteria were grown in SP4 broth, concentrated by centrifugation and fixed with 2.5% glutaraldehyde in 0.1 M phosphate buffer. Figures 2A and 2B show the thick sections for concentrated samples of the two different bacteria embedded in epoxy resin. NBIMC_P1-C2 microbes appeared to be more slender and pointed bacteria. In comparison, many of the rod-shaped NBIMC_P2-C1 microbes looked slightly larger or thicker. The sections of both NBIMC_P1-C2 and NBIMC_P2-C1 revealed occasional microbes that appeared to be more pleomorphic in shape and larger in size. The ultra-thin sections revealed unique ribosomal structures, occasional electron-dense bodies, fine nucleic acid structures and differences in bacterial wall structures of the 2 different microbes obtained from the 1^st^ and the 2^nd^ phases of growth in the culture (Figures 2C and 2D). Atypical outer membranes of Gram-negative bacterial wall structures were seen in some NBIMC_P2-C1 microbes (Figure 2D). The more round and pleomorphic microorganisms had less-rigid and noticeably wavy outer membranes in their wall structures (Figure 2E). 

### Whole genome sequencing of isolates from the 1^st^ and the 2^nd^ phases of microbial growth in broth culture

Whole-genome sequencing of single colony-derived isolates cloned from the 1^st^ phase (NBIMC_P1-C1, P1-C2 and P1-C3) and the 2^nd^ phase (NBIMC_P2-C1, P2-C2, P2-C3 and P2-C4) of microbial growths in the blood culture was conducted using the Illumina MiSeq platform (Table 2). The raw reads generated from each bacterial isolate in genomic sequencing were first assembled into contigs using CLC Bio Genomics Workbench bioinformatic tool. 

**Table 2:** Whole genome sequencing datasheet of single-colony cloned microbes from the 1st phase (P1) and the 2nd phase (P2) microbial growth in NBIMC blood culture.

		**NBIMC_P1**			**NBIMC_P2**				
	**C1 clone**	**C2 clone**	**C3 clone**		**C1 clone**	**C2 clone**	**C3 clone**	**C4 clone**	
**No. of raw reads**	6,759,170	4,842,336	4,166,696		9,200,992	9,024,618	17,221,946	11,971,358	
**Total reads length (bp)**	798,667,429	768,566,565	706,133,276		1,789,380,704	1,728,394,985	4,074,252,920	2,847,032,354	
**No. of qualified reads**	6,716,274	4,747,508	4,135,906		9,174,760	8,983,874	17,219,778	11,969,868	
**No. of reads in contigs**	6,646,354	4,685,007	4,093,548		9,087,368	8,855,687	16,787,708	11,387,242	
**No. of contigs formed**	89	66	69		92	85	44	60	
**Max contig length**	402,855	548,545	548,545		345,443	786,056	991,132	990,730	
**N50**	124,487	223,703	166,412		166,173	182,430	280,857	207,500	
**Total contig length**	4,993,310	4,996,867	4,996,806		5,948,199	5,536,659	5,542,402	5,951,317	
**Estimated coverage**	160	154	141		300	311	735	476	
**GC contents (%)**	61.5	61.5	61.5		63.1	63.4	63.4	63.1	

#### (1): Whole genome sequencing analysis of the 1^st^ phase growth isolates

The genomic sequencing result revealed total contig lengths of formed contigs from all 3 single-colony cloned isolates of the 1^st^ phase microbial growth were ~ 5 million base pairs (Mb) (Table 2). The GC content of the presumed ~ 5 Mb microbial genome was 61.5%. The sequences obtained for each of the 3 isolates had the average of 141 to 160-fold coverage of the draft genomes.

**Figure 3:**
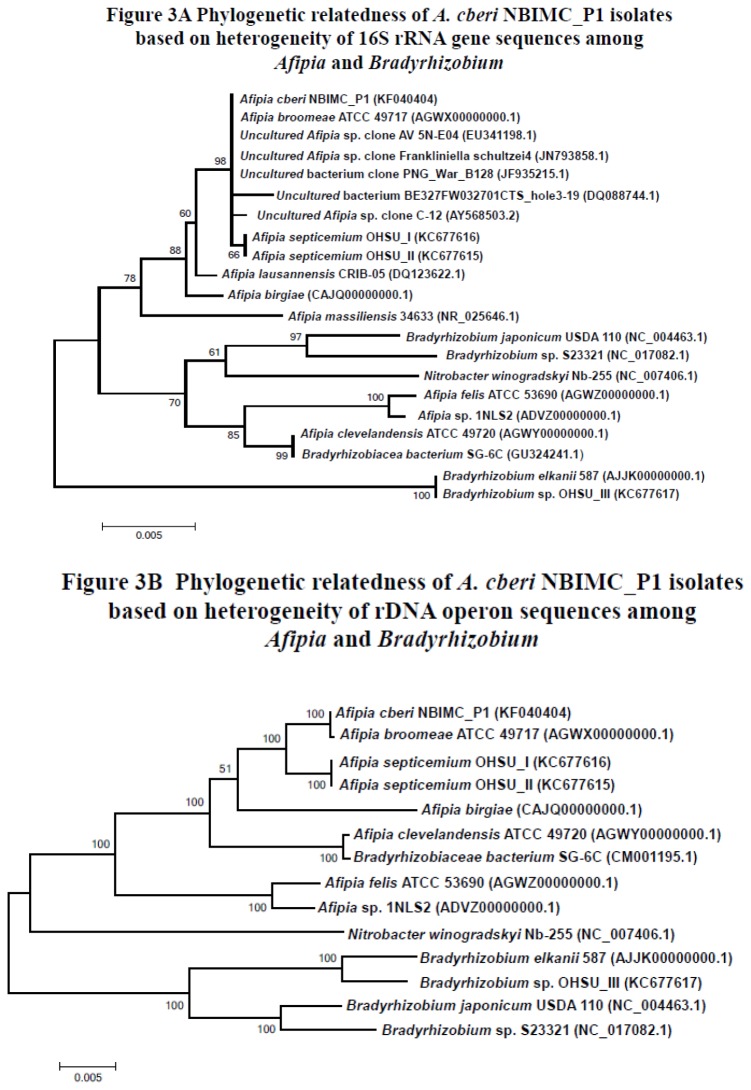
Phylogenetic relatedness of *Afipia cberi* NBIMC_P1 among *Afipia* sp. and *Bradyrhizobium* sp. microbes. Phylogenetic analysis for NBIMC_P1 isolates based on 16S rRNA gene sequences (**A**) and whole rDNA operon sequences (**B**) using the neighbor-joining method. GenBank Accession numbers of sequences used in the analyses are shown in parentheses. Scale bar units are estimated branch lengths. Numerals indicate bootstrap percentages over 50 after 500 replications.

**Analysis of 16S rRNA gene and rDNA operon sequences:**The sequences of 16S rRNA gene (~ 1.4 Kb) as well as the whole rDNA operon (~ 5.4 Kb) from the 3 single-colony cloned isolates from the initial phase of growth (NBIMC_P1) were found to be identical. Comparison of the 16S rRNA gene and whole rDNA operon sequences with those in the NCBI database showed the 3 single–colony-cloned isolates of NBIMC_P1 were microbes in the Family of *Bradyrhizobiaceae*, closely related to those of *Afipia* species (Figure 3 A and B). The microbes in the genus and even the family are known to have very high levels of homology in their 16S rRNA gene sequences. As such, these sequences are not good indicators to determine species or genera diversity [12,15]. Since all *Bradyrhizobiaceae* have their 3 rRNA genes similarly organized and co-transcribed as an operon, analysis based on the variations in sequences of 5.4 Kb rDNA operons could be much more informative in the study of highly challenging *Bradyrhizobiaceae* taxonomy [9]. Analysis of the rDNA operon sequences revealed that NBIMC_P1 isolates are phylogenetically most closely related to *A. broomeae*, followed by our recently isolated *A. septicemium* [9]. They are more distant from *A. clevelandensis, A. birgiae* and *A. massiliensis*, and most distant from *A. felis* (Figure 3B). 

**Figure 4:**
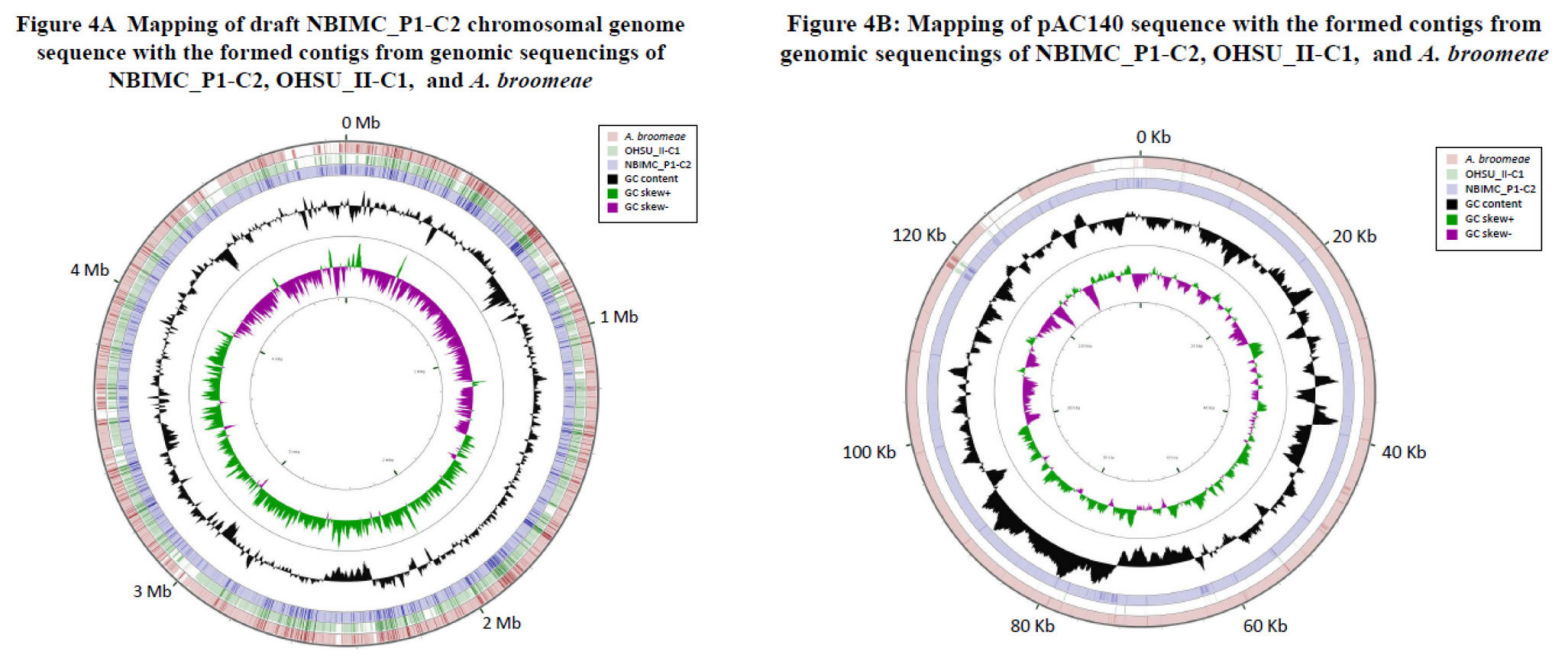
Sequence mapping for the draft genome of *Afipia cberi* NBIMC_P1 and its circular plasmid pAC140. (A) Sequence mapping for the draft genome of *A. cberi* NBIMC_P1-C2 against those of *A. septicemium* OHSU_II-C1 and *A. broomeae*. The tracks from inside to outside represent GC skews, GC contents, draft genome of NBIMC_P1-C2 microbe, draft genome of OHSU_II-C1 microbe and *A. broomeae* genome. The color blank regions represent sequence differences found between the bacterial genomes. Major regions of difference are seen in the regions of 0.9-1.1 Mb, 2.6 Mb and 4.8-4.9 Mb. (B) Sequence mapping for the circular plasmid pAC140 against the formed contigs of NBIMC_P1-C2, *A. septicemium* OHSU_II-C1 and *A. broomeae*. The tracks from inside to outside represent GC skews, GC contents, formed contigs with draft genome of NBIMC_P1-C2 microbe, OHSU_II-C1 microbe and *A. broomeae*. The track of *A. septicemium* OHSU_II-C1 is blank with no pAC140-related sequences found in formed contigs of the sample. In contrast, a large portion of pAC140 –related sequence can be mapped with the reported supercontig 1.4 (128 Kb) of *A. broomeae* [17], except 2 specific regions of 123 to 128 Kb and 135 Kb to 140 Kb.

**Analysis of genome sequences:** We constructed draft genomes for *A. cberi* strains NBIMC_P1-C1, P1-C2 and P1-C3 by assembling the formed contigs using CONTIGuator tool [10] as described in our recent publication [9]. The complete genome of *Bradyrhizobiaceae bacterium* strain SG-6C [16] and the working complete genome of *A. broomeae* [17] served as the references. Figure 4A shows sequence mapping of closely related NBIMC_P1-C2, *A. septicemium* and *A. broomeae* genomes with regions of difference identified using CGView program [18]. Blank color regions of marked sequence difference could clearly be identified between genomes of NBIMC_P1-C2 and *A. septicemium* as well as between genomes of NBIMC_P1-C2 and *A broomeae*. Comparison for the similarity or dissimilarity of genome content among draft genomes of different isolates NBIMC_P1-C1, C2 and C3, and those of *A. septicemium* as well as other 5 established *Afipia* *sp.* was conducted by using the informatics tool progressiveMauve [11]. The genome comparison revealed the 3 single-colony clones of NBIMC_P1-C1, C2 and C3 were highly similar to each other in their genome content (GC content of 61.5%), with a dissimilarity value less than 0.2% (Table 3). The NBIMC_P1 isolates appeared to be most closely related to *A. broomeae*, but the 2 microbes still had ~ 12% of dissimilarity in their respective genome content. The NBIMC_P1 isolates should represent a new *Afipia* species, tentatively named *A. cberi* in this study. In comparison, *A. cberi* NBIMC_P1 isolates and the recently discovered strains OHSU_I and OHSU_II of *A. septicemium* had ~ 23% dissimilarity in genome content. There is significantly higher dissimilarity (~ 30%) in genome content between *A. cberi* and *A. clevelandensis* or *A. birgiae*. The isolates of *A. cberi* are genetically most distant (~ 45%) from *A. felis*.

**Table 3:** Genome content differences among *Afipia cberi* NBIMC_P1 isolates, established *Afipia* species, *Afipia septicemium* OHSU_I-C4, OHSU_II-C1 and *Bradyrhizobiaceae* SG.

	**1**	**2**	**3**	**4**	**5**	**6**	**7**	**8**	**9**	**10**	**11**
**1. *A. cberi* NBIMC_P1-C1**	-	0.16%	0.10%	22.81%	22.83%	11.95%	30.04%	29.99%	44.59%	43.43%	29.90%
**2. *A. cberi* NBIMC_P1-C2**	0.16%	-	0.09%	22.82%	22.84%	11.96%	30.06%	30.00%	44.61%	43.44%	29.93%
**3. *A. cberi* NBIMC_P1-C3**	0.10%	0.09%	-	22.79%	22.81%	11.92%	30.03%	29.98%	44.59%	43.43%	29.90%
**4. *A. septicemium* OHSU_I-C4**	22.81%	22.82%	22.79%	-	0.03%	22.38%	30.43%	30.63%	44.88%	43.21%	30.34%
**5. *A. septicemium* OHSU_II-C1**	22.83%	22.84%	22.81%	0.03%	-	22.45%	30.45%	30.65%	44.89%	43.23%	30.36%
**6. *A. broomeae***	11.95%	11.96%	11.92%	22.38%	22.45%	-	31.16%	30.66%	45.10%	44.05%	30.89%
**7. *A. clevelandensis***	30.04%	30.06%	30.03%	30.43%	30.45%	31.16%	-	32.22%	44.92%	43.89%	10.14%
**8. *A. birgiae***	29.99%	30.00%	29.98%	30.63%	30.65%	30.66%	32.22%	-	45.31%	44.46%	32.16%
**9. *A. felis***	44.59%	44.61%	44.59%	44.88%	44.89%	45.10%	44.92%	45.31%	-	27.01%	44.74%
**10. *Afipia* sp. 1NLS2**	43.43%	43.44%	43.43%	43.21%	43.23%	44.05%	43.89%	44.46%	27.01%	-	43.73%
**11. *Bradyrhizobiaceae* SG**	29.90%	29.93%	29.90%	30.34%	30.36%	30.89%	10.14%	32.16%	44.74%	43.73%	-

Rows 1-11 correspond to columns 1-11.

Our genomic sequencing analysis and comparison among NBIMC_P1 isolates and established *Afipia* sp. revealed the 3 single-colony cloned *A. cberi* NBIMC_P1 isolates carry a ~ 140 Kb circular plasmid, pAC140. The circular plasmid could not be found in other established *Afipia* sp. and not in *A. septicemium* OHSU_I or OHSU_II isolates (Figure 4B). However, its sequence was found to have high homology with a ~ 128 Kb gene segment designated previously as supercontig 1.4 in a reported *A. broomeae* draft genome [17]. As described in our previous study [9], this particular gene segment of *A. broomeae* was suspected to be a plasmid, because it revealed significant homology with several plasmids of alpha-2 proteobacteria in Blastn search against GenBank database. The *A. cberi* plasmid pAC140 (GC content of 62.6%) has coding regions of 120,609 bp (86.3 % of the plasmid) with no RNA gene, 155 predicted protein-coding genes and 89 proteins with known functions. But, analysis showed no identifiable antibiotic resistant gene. Sequence comparison of pAC140 with *A. broomeae* supercontig 1.4 shows that pAC140 carries additional 2 gene segments with overall ~ 9.5 Kb sequences that are not present in supercontig 1.4, the presumed plasmid of *A. broomeae* (Figure 4B). Blastn search revealed the 2 gene segments had homology with putative ABC transporter and integrase-transposase genes. 

#### (2): Whole genome sequencing analysis of the 2^nd^ phase growth isolates

The total contig lengths of formed contigs from all 4 single-colony cloned isolates of the 2^nd^ phase microbial growth (NBIMC_P2-C1, P2-C2, P2-C3 and P2-C4) were ~ 5.5 Mb or ~ 5.9 Mb (Table 2). Two clones with the presumed genome size of ~ 5.9 Mb had GC content of 63.1% and the other 2 clones with the presumed genome size of ~ 5.5 Mb had GC content of 63.4%. The sequences obtained for each of the 4 isolates had the average of 300 to 735-fold of the presumed genome coverage.

**Figure 5:**
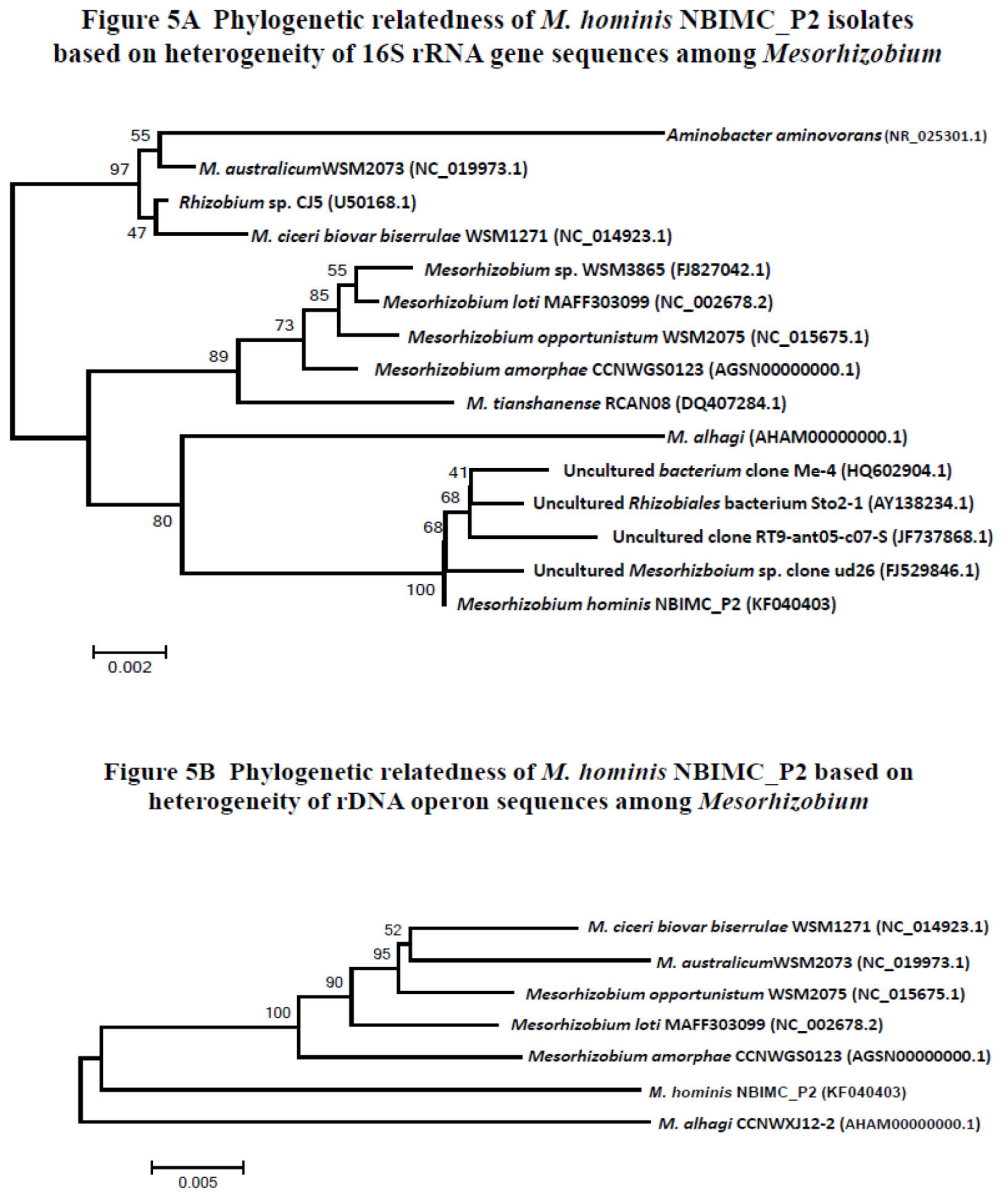
Phylogenetic relatedness of *Mesorhizobium hominis* NBIMC_P2 among *Mesorhizobium* sp. microbes. Phylogenetic analysis for NBIMC_P2 isolates based on 16S rRNA gene sequences (**A**) and whole rDNA operon sequences (**B**) using the neighbor-joining method. GenBank Accession numbers of sequences used in the analyses are shown in parentheses. Scale bar units are estimated branch lengths. Numerals indicate bootstrap percentages over 50 after 500 replications.

**Analysis of 16S rRNA gene and rDNA operon sequences:** Sequencing results showed all 4 single-colony cloned isolates from the 2^nd^ phase growth (NBIMC_P2) had the identical 16S rRNA gene and the whole rDNA operon sequences. Phylogenetic comparison of the sequences of NBIMC_P2 isolates with those deposited in the NCBI database revealed the isolates are a previously unknown species of bacteria related to those of *Mesorhizobium* in the Family of *Phyllobacteriaceae* (Figure 5). Figure 5A shows the phylogenetic tree based on heterogeneity in sequences of 16S rRNA genes submitted to NCBI. Figure 5B shows the phylogenetic analysis of sequences of rDNA operons among these isolates and established species of *Mesorhizobium* with their genomic sequences available in NCBI. We tentatively named the NBIMC_P2 isolates, the first *Mesorhizobium* isolated from humans, *Mesorhizobium hominis* in this study.

**Figure 6:**
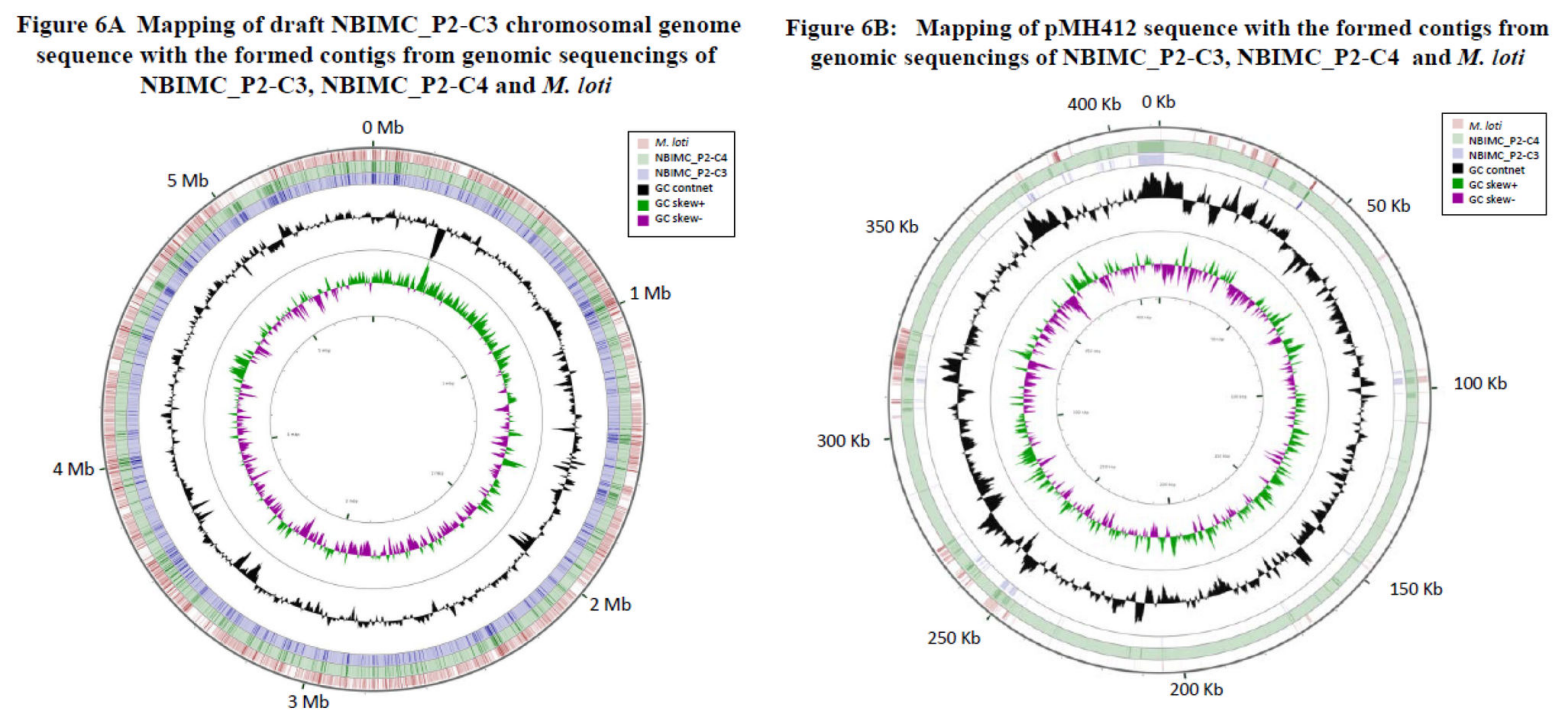
Sequence mapping for the draft genome of *Mesorhizobium hominis* NBIMC_P2 and its circular plasmid pMH412. (A) Sequence mapping for the draft genome of *M. hominis* NBIMC_P2-C3 against those of *M. hominis* NBIMC_P2-C4 and complete genome of *M. loti* including 2 plasmids pMLa and pMLb. The tracks from inside to outside represent GC skews, GC contents, draft genome of NBIMC_P2-C3 microbe, draft genome of NBIMC_P2-C4 microbe and *M. loti* genome. The color blank regions represent sequence differences found between the bacterial genomes. Major regions of difference are seen in the regions of 0.4-0.5 Mb, 0.6-0.7 Mb, 1.8-1.9 Mb, 2.3-2.4 Mb and 3.1-3.2 Mb. (B) Sequence mapping for the circular plasmid pMH412 against the formed contigs of NBIMC_P2-C3, NBIMC_P2-C4 and complete genome of *M. loti* genome including pMLa and pMLb. The tracks from inside to outside represent GC skews, GC contents, formed contigs with draft genome of NBIMC_P2-C3, NBIMC_P2-C4 and *M. loti* complete genome including pMLa and pMLb. Most of the tracks representing formed contigs of NBIMC P2-C3 and *M. loti* genome including pMLa and pMLb are blank. Sequence of pMH412 can only be mapped with the formed contigs from NBIMC_P2-C4, not with the formed contigs from NBIMC_P2-C3 or *M. loti* complete genome with pMLa and pMLb.

**Analysis of genome sequences:** We constructed draft genomes for all the 4 single-colony-cloned NBIMC_P2 isolates of *M. hominis* using the complete genome of *M. loti* as reference [17]. An apparent difference in total length of the formed contigs from genomic sequencing of these 4 single-colony cloned isolates was noticed (Table 2). The difference (~ 5.94 Mb of NBIMC_P2-C1 and P2-C4 versus ~ 5.54 Mb of NBIMC_P2-C2 and P2-C3) was found to be the result of presence or absence of a circular 412.1 Kb plasmid, pMH412. The *M. hominis* plasmid pMH412 has a GC content of 59.7%, while the finished draft genomes of *M. hominis* NBIMC_P2 isolates have a GC content of 63.3%. Isolates of NBIMC_P2-C1 and NBIMC_P2-C4 apparently possess pMH412 while isolates of NBIMC_P2-C2 and NBIMC_P2-C3 do not. Figure 6A shows sequence mapping for draft genomes of *M. hominis* NBIMC_P2-C3 with total length of the formed contigs ~ 5.5 Mb), *M. hominis* NBIMC_P2-C4 (with total length of the formed contigs ~ 5.9 Mb) and *M. loti* with regions of difference identified using CGView program. There is no region with sequence difference identified between the chromosomal genomes of NBIMC_P2-C3 and NBIMC_P2-C4, *M. hominis* isolates (Figure 6A). In comparison, the color blank regions with marked sequence difference could readily be identified between the chromosomal genome of *M. hominis* NBIMC_P2-C3 and that of *M. loti* containing 2 different pML plasmids. Figure 6B shows sequence mappings of pMH412 against the formed contigs from total genomic sequencing reads of NBIMC_P2-C4 and NBIMC_P2-C3 as well as *M. loti* genome that possesses 2 plasmids, pMLa (351,911 bp; GenBank accession number NC_002679.1) and pMLb (208315 bp; GenBank accession number NC_002682.1) [19]. The result in Figure 6B indicates the sequence of pMH412 is present only in the formed contigs of NBIMC_P2-C4, not in those of NBIMC_P2-C3 and *M. loti*. There is no sequence homology found between pMH412 and the 2 plasmids of *M. loti*. 

The pMH412 has coding regions of 362824 bp (88% of the 41214 bp plasmid) with no RNA gene, 423 predicted protein-coding genes and 276 proteins with known functions. Analysis showed no identifiable antibiotic resistant gene. Continual growth of the plasmid-containing isolates/clones of *M. hominis* in culture would apparently result in quickly losing the plasmid. Many of the single-colony clones picked from the early stage of NBIMC_P2-C1 and NBIMC_P2-C4 cultures still possessed the plasmid pMH412.1. However, the plasmid was apparently lost in the subsequent single-colony clones picked from the NBIMC_P2-C1 and NBIMC_P2-C4 cultures after couples of passages. The new clones no longer maintained the plasmid pMH412.

Comparison for the similarity or dissimilarity of contents among draft chromosomal genomes of the 4 single-colony-cloned isolates of *M. hominis* and those of established *Mesorhizobium* sp. was conducted by using informatics tool progressiveMauve. The comparison revealed the contents of genomes for 4 single-colony cloned isolates from NBIMC_P2 were highly similar to one another, with dissimilarity less than 0.2% (Table 4). However, genome contents of *M. hominis* isolates had a dissimilarity of ~ 62.3 % with that of *M. loti*. They also had very high dissimilarity (62 to 68.3%) with those of other established *Mesorhizobium*, including *M. opportunistum, M. amorphae, M. australicum* and *M. alhagi* (Table 4). 

**Table 4:** Genome content differences among NBIMC_P2 isolates and established *Mesorhizobium* species.

	**1**	**2**	**3**	**4**	**5**	**6**	**7**	**8**	**9**	**10**
**1. *M. hominis* NBIMC_P2-C1**	-	0.13%	0.13%	0.14%	62.28%	62.28%	62.48%	62.00%	68.30%	62.62%
**2. *M. hominis* NBIMC_P2-C2**	0.13%	-	0.14%	0.14%	62.27%	62.28%	62.47%	62.00%	68.31%	62.64%
**3. *M. hominis* NBIMC_P2-C3**	0.13%	0.14%	-	0.10%	62.29%	62.29%	62.48%	62.00%	68.31%	62.64%
**4. *M. hominis* NBIMC_P2-C4**	0.14%	0.14%	0.10%	-	62.29%	62.30%	62.48%	62.01%	68.32%	62.65%
**5. *M. loti***	62.28%	62.27%	62.29%	62.29%	-	39.86%	41.28%	43.12%	67.11%	48.98%
**6. *M. opportunistum***	62.28%	62.28%	62.29%	62.30%	39.86%	-	34.05%	35.06%	67.08%	49.96%
**7. *M. australicum***	62.48%	62.47%	62.48%	62.48%	41.28%	34.05%	-	35.61%	67.46%	51.07%
**8. *M. ciceri***	62.00%	62.00%	62.00%	62.01%	43.12%	35.06%	35.61%	-	66.89%	49.47%
**9. *M. alhagi***	68.30%	68.31%	68.31%	68.32%	67.11%	67.08%	67.46%	66.89%	-	66.13%
**10. *M. amorphae***	62.62%	62.64%	62.64%	62.65%	48.98%	49.96%	51.07%	49.47%	66.13%	-

Rows 1-10 correspond to columns 1-10.

### Antibiotic susceptibility study

We conducted an *in vitro* antibiotic susceptibility study for the newly discovered microbes of *A. cberi* and *M. hominis* using broth culture system with serial dilutions of antibiotics [12]. The study revealed microbial isolates of *A. cberi* were susceptible to penicillin G, cephalothin, ceftriaxone, cefoxitin, rifampicin and streptomycin based on the minimal inhibition concentrations (MIC) of these antibiotics. Our recently discovered *A. septicemium* OHSU_I and OHSU_II strains were also found to be susceptible to these antibiotics, but not to streptomycin. However, microbial isolates of *M. hominis* with or without the presence of plasmid pMH412.1 were not susceptible to nearly all the antibiotics tested including the antibiotics commonly used to treat intracellular bacterial infections such as doxycycline and tetracycline (Table 5). Only the MIC of cefoxitin indicated microbial isolates of *M. hominis* could be considered marginally sensitive to the antibiotic. *Escherichia coli* (*E. coli*) ATCC 25922 and *Staphylococcus aureus* (*S. aureus*) ATCC 29213 were studied in parallel as the quality control for expected potency of the testing antibiotics against the drug-sensitive bacteria.

**Table 5:** Antibiotic susceptibility study for microbial isolates of *Afipia* species and *Mesorhizobium* species.

**Antibiotics**	**Bacteria**									
	***Afipia cberi***		Afipia septicemium*		***Mesorhizobium hominis***				***E. coli***	***S. aureus***
	NBIMC_P1-C1	NBIMC_P1-C2	OHSU_I-C4	OHSU_II-C2	NBIMC_P2-C1	NBIMC_P2-C2	NBIMC_P2-C3	NBIMC_P1-C4	ATCC 25922	ATCC 29213
**Penicillin G**	2	2	2	1	64	64	128	64	32	0.25
**Amoxicillin**	64	64	64	64	64	64	64	64	2	0.5
**AMC**	1	1	1	1	64	64	64	64	2	0.5
**Ampicillin**	8	8	8	8	16	8	8	64	2	0.5
**Piperacillin**	4	4	8	8	128	128	128	64	4	1
**Cefoxitin**	1	1	2	1	4	8	8	4	8	2
**Cephalothin**	0.25	0.25	0.5	0.5	64	64	64	64	8	0.12
**Ceftriaxone**	0.5	0.5	0.5	0.5	16	64	128	16	0.25	1
**Imipenem**	8	8	8	8	16	16	16	32	0.12	0.03
**Amikacin**	8	8	8	8	32	64	64	64	2	4
**Kanamycin**	8	8	8	8	128	128	128	128	4	8
**Gentamicin**	32	32	16	16	8	8	8	8	4	8
**Streptomycin**	4	4	64	32	>128	>128	>128	>128	1	4
**Tetracycline**	8	8	32	32	8	8	16	8	0.5	0.12
**Doxycycline**	8	8	32	32	16	16	32	32	0.5	<0.12
**Ciprofloxacin**	4	4	16	16	8	4	8	8	0.12	0.25
**Levofloxacin**	8	8	32	32	16	16	16	16	0.03	0.03
**Rifampicin**	2	2	2	1	16	4	4	4	4	0.015
**Erythromycin**	32	32	128	128	>128	>128	>128	>128	128	1
**TMP-SMX**	32	32	32	32	64	64	64	64	2	2
**Vanomycin**	64	64	>128	>128	32	32	32	32	>128	0.5

Values are MIC (mg/l). AMC: Amoxicillin/Clavulanic acid (2:1), TMP-SMX: Trimethoprim/sulfamethoxazole (1:5). The results of AMC and TMP-SMX are the MICs of Amoxicillin and Trimethoprim.

^*^ The isolation and characterization of *A. septicemium* was described in Ref #9.

## Discussion

In this study we describe finding a mixed infection by 2 different *Rhizobiales* in the blood sample culture broth from a patient who succumbed to a fulminant course of progressive lung disease in 1999. A 2^nd^ phase of microbial growth (NBIMC_P2) was observed nearly 2 months later after the 1^st^ phase of microbial growth (NBIMC_P1) occurred. In comparison with *A. cberi* isolated from the initial phase of growth, NBIMC_P2 microbes apparently stayed longer in an inactive state, but grew faster and could reach into a significantly higher cell density plateau in the microbial culture system once they underwent proliferation. Genomic characterization demonstrated they are novel *Phyllobacteriaceae*, phylogenetically most related to those of *Mesorhizobium*. This is evidently the first *Mesorhizobium*-related microbe isolated from humans, thus the NBIMC_P2 isolates are tentatively referred to as strains of *M. hominis* in this study. However, there are marked sequence heterogeneities of 16S rRNA genes and rDNA operons as well as significant differences in genome contents between *M. hominis* NBIMC_P2 and established *Mesorhizobium* species (Figure 5 and Table 4). *M. hominis* could prove to be microbes that are separated taxonomically from the Genus of *Mesorhizobium* after further studies. 

Genomic studies revealed all 3 isolates or clones of *A. cberi* possess a previously unknown stable circular ~ 140 Kb plasmid, pAC140. No plasmid has previously been reported in any established species of *Afipia*. However, as described in our previous study [9], the reported supercontig 1.4 of *A. broomeae* [17] would likely represent an unrecognized plasmid. Sequence comparison has identified 2 additional gene segments with total ~ 9.5 Kb sequences in pAC140, evidently not present in supercontig 1.4, the presumed plasmid, or in the entire formed contigs of *A. broomeae* (Figure 4B). Detail of *A cberi* genome and plasmid structures will be presented separately. Interestingly, genomic sequencing study of *M. hominis* obtained from the 2^nd^ phase microbial growth in culture revealed that some clones/isolates also had a circular ~ 412.1 Kb plasmid, pMH412. However, clones of *M. hominis* tested positive for pMH412 were found to quickly lose the plasmid when the bacteria were kept for prolonged culturing or undergoing continual cell passage. The instability of extrachromosomal plasmids was reported previously in some of the *Rhizobial* microbes [20,21]. The significance in biology and in clinical implications of the 2 plasmids identified in *A. cberi* and *M. hominis* is not clear at this moment. The possible virulence effects of these plasmids including antibiotics susceptibilities of the bacteria and their potential pathogenic association with infections in humans will require further studies.

Testing of antibiotic susceptibility for *A. cberi* and *M. hominis* isolates revealed the microbes particularly those of *M. hominis* appear to be resistant to most of the commonly used antibiotics (Table 5). Since *Afipia, Bradyrhizobium* and *Mesorhizobium* are a group of microbes that are potentially able to invade, survive and grow in eukaryotic cells, antibiotic susceptibility results from the tests conducted in cell-free microbial cultures against these facultative intracellular microbes may not be applicable clinically to the treatment of infected patients. Development of a better drug sensitivity assay system that is more relevant to the *in vivo* condition of intracellular infections by these microbes will be more ideal. However, for *M. hominis* that are not susceptible to nearly all the antibiotics tested in a cell-free microbial culture system, they are likely to be even less sensitive to the antibiotics if they have invaded and survived inside the eukaryotic host cells.

The bacteria in the Subclass of alphaproteobacteria that are known to be pathogenic to humans are those in the Orders of *Rickettsiales* and *Rhizobiales*. In the Order of *Rhizobiales*, members in the Families of *Brucellaceae* and *Bartonellaceae* [22,23], some *Afipia* species of *Bradyrhizobiaceae* [24] and *Rhizobium* (Agrobacterium) *radiobacter* of *Rhizobiaceae* [25–27] infect humans and produce various acute and chronic forms of illnesses. However, most members of *Bradyrhizobium* in the Family of *Bradyrhizobiaceae* and members of *Mesorhizobium* in the Family of *Phyllobacteriaceae* were generally known as soil bacteria [28]. On the other hand, microbes in this unique group of *Rhizobiales* are facultative intracellular microbes that could effectively invade and grow in eukaryotic host cells [29]. The symbiotic *Bradyrhizobium* and *Mesorhizobium* microbes utilize amino acids from plant cells to survive and grow, but provide the infected plants with much needed fixed-form of nitrogen by taking atmosphere free nitrogen and converting it into ammonia or ammonium [30]. It is important to note that none of the nif and nod genes or symbiosis Islands found in genomes of symbiotic nitrogen-fixation bacteria isolated from soil or plants are identified in the newly discovered *Bradyrhizobium* species OHSU_III [8] and *M. hominis*. However, similarities between the strategies adopted by pathogenic and symbiotic *Rhizobiales* in infecting the eukaryotic hosts have been described [29]. 

The findings of infections, in addition to new *Afipia* microorganisms, by novel microbes of *Bradyrhizobium* and *Mesorhizobium* in humans reveal a previously unrecognized host spectrum as well as nature of infections by microbes of *Bradyrhizobiaceae* and *Phyllobacteriaceae* Families in *Rhizobiales*. In this context, a recent sequences-based discovery of new *Bradyrhizobium enterica* in the biopsies of cord blood transplanted patients with cord colitis syndrome [31] is important in revealing infections of the otherwise unknown and uncultured *Bradyrhizobiaceae* bacteria in patients. The analysis of 16 S rRNA gene r-RNA operon sequences shows that *Bradyrhizobium enterica* and our recent culture-based identification of new *Bradyrhizobium* sp. OHSU-III [9] are phylogenetically closely related. Comparison of genome sequences (NCBI accession numbers: AMFB00000000 and APJD00000000) using progressiveMauve shows the 2 new *Bradyrhizobium* microbes found infecting human hosts are more closely related to each other than to any other established species of *Bradyrhizobium* microbes. However, the 2 *Bradyrhizobium* are apparently different and possess ~ 30% dissimilarities between their genome contents. More novel species of *Bradyrhizobiaceae* and *Phyllobacteriaceae* microbes will likely be found infecting human hosts*.*

Importantly, isolations of these microbes from blood samples indicates these newly discovered *Rhizobiales*, like those human pathogenic microbes of *Brucellaceae, Bartonellaceae* and *Rhizobiaceae* Families in the *Rhizobiales*, could be related to hematogenous infections and could be disseminated systemically in the infected patients. Also significantly, the study has demonstrated that a patient could apparently be infected at the same time by a combination of 2 different *Rhizobiales* microbes. However, it is important to stress that it is presently unclear what the roles of these 2 newly discovered *Rhizobiales* played, if any, in this patient’s unrelenting respiratory distress illness. Follow-up studies including re-examination of the diseased lung tissues from patient’s autopsy could better unveil the possible role in the necrotizing process of infected lung for each of the microbe isolated. Successful growth and isolation of these previously unknown and uncultivated microbes by culture has made it possible to analyze metabolic and pathogenicity roles of the microbes in various chronic or acute forms of human disease processes. Many important questions including prevalence of infections, mode of transmission and diseases association can only be answered through further studies and development of better microbial, molecular, and serological assays against the newly identified microbes of *Rhizobiales*. 
